# Examining the cognitive processes underlying resumption costs in task-interruption contexts: Decay or inhibition of suspended task goals?

**DOI:** 10.3758/s13421-023-01458-8

**Published:** 2023-09-06

**Authors:** Patricia Hirsch, Luca Moretti, Sibel Askin, Iring Koch

**Affiliations:** Cognitive and Experimental Psychology, Institute of Psychology, RWTH Aachen University, Jägerstr. 17-19, D-52066 Aachen, Germany

**Keywords:** Task interruptions, Resumption costs, Inhibition, Persisting activation

## Abstract

To examine whether an ongoing primary task is inhibited when switching to an interruption task, we implemented the *n* − 2 backward inhibition paradigm into a task-interruption setting. In two experiments, subjects performed two primary tasks (block-wise manipulation) consisting of a predefined sequence of three subtasks. The primary tasks differed regarding whether the last subtask switched or repeated relative to the penultimate subtask, resulting in *n* − 1 switch subtasks (e.g., AB*C*) and *n* − 1 repetition subtasks (e.g., AC*C*) as the last subtask of the primary task. Occasionally, an interruption task was introduced before the last subtask of a primary task, changing the last subtask of the primary task from a *n* − 1 switch subtask to a *n* − 2 switch subtask (e.g., A*B* → secondary task → *C*) and from a *n* − 1 repetition subtask to a *n* − 2 repetition subtask (e.g., A*C* → secondary task → *C*). In two experiments with different degrees of response-set overlap between the interruption task and the subtasks of the primary task, we observed that switching back from the interruption task to the primary task resulted in *n* − 2 switch costs in the first subtask after the interruption (i.e., worse performance in *n* − 2 switch subtasks than in *n* − 2 repetition subtasks). This *n* − 2 switch cost was replicated in a third experiment in which we used a predefined sequence of four subtasks instead of three subtasks. Our finding of *n* − 2 switch costs suggest that the last subtask performed before the interruption remains activated when switching to the interruption task.

In everyday life, we are often confronted with task-interruption demands which typically have a harmful effect on the performance in the interrupted task (see, e.g., Hirsch et al., 2022; Trafton & Monk, 2007; Werner et al., 2015, for reviews). The present study focuses on the cognitive mechanisms underlying this performance decline. The understanding of the cognitive basis of the performance deterioration in interrupted tasks informs theories about the basic operating principles of human cognition, and these theories can help to develop practical recommendations for task-environments and task-interruption management which may reduce the disruptive effects of task interruptions in applied settings.

## Task-interruption costs

In task-interruption situations, an ongoing task, termed primary task, is temporarily suspended in order to perform a new task, referred to as secondary task (e.g., Brixey et al., 2007; Trafton et al., 2003). In contrast to distractions, which solely require us to ignore task-irrelevant information (e.g., to notice a loud conversation during the preparation of a medication administration), task interruptions are accompanied by the requirements of forming the intention to resume the primary task, encoding the processing state of the primary task (i.e., to facilitate the resumption of the primary task), and dealing with an additional task (e.g., Clapp & Gazzaley, 2012; Grundgeiger et al., 2010).

It is well-known that task interruptions have several adverse effects on the performance in the primary task (for reviews, see, e.g., Couffe & Michael, 2017; Hirsch et al., 2022). The primary task is, for instance, often forgotten to be resumed; and if the primary task is resumed, typically, more time is needed for the completion of the task and more errors are made than in noninterrupted tasks (e.g., Altmann et al., 2014; Bailey & Konstan, 2006; Dodhia & Dismukes, 2009; Lee & Duffy, 2015; Li et al., 2008; Monk et al., 2004).

Numerous studies have measured the resumption lag as a behavioural marker of the harmful effect of task interruptions on primary task performance. The resumption lag is the temporal interval between the last response in the secondary task and the first response in the resumed primary task. A typical finding of these studies is that the resumption lag is longer than the time lag between two consecutive responses in a noninterrupted primary task (e.g., Blumberg et al., 2015; see also Salvucci, 2013). This performance decline in the interrupted tasks relative to noninterrupted primary tasks reflects resumption costs.

Task interruptions not only prolong the process of selecting the next step in a primary task after an interruption, but they also increase the vulnerability to errors during the resumption process (Altmann et al., 2017). To explore the effect of task interruptions on resumption errors (i.e., errors in the first step back at the primary task), many studies have employed procedural tasks as primary tasks. Procedural tasks consist of multiple subtasks (i.e., two-choice response time tasks such as categorizing a digit as odd or even) that have to be performed in a predefined order without skipping or repeating a subtask.

Procedural tasks can be contrasted with continuous tasks that require a continuous stream of actions over a longer time period, such as car driving (e.g., Salvucci, 2005). Thus, continuous primary tasks are well suited to examine strategies in task-interruption management. In contrast, procedural tasks consist of clearly identifiable subtasks. The subtasks has a definitive observable start and end point (i.e., stimulus onset and response execution), allowing us to analyze performance based on a fine-grained subtask level. Since procedural tasks comprise several subtasks which have to be performed in a predefined order, they also allow for the differentation between nonsequence errors and sequence errors (e.g., Altmann et al., 2017; see also Moretti et al., 2022).

A nonsequence error occurs, when the correct subtask in a predefined sequence of subtasks is selected but incorrectly executed. In contrast, in a sequence error, a wrong subtask in the predefined sequence of subtasks is selected but correctly executed (i.e., repeating or ommitting a step). Whereas nonsequence errors are thought to rely on general attentional resources, sequence errors are assumed to rely on memory processes (Altmann et al., 2017).

## Cognitive basis of task-interruption costs

Attention plays an essential role in the control of actions. In task-interruption situations, we suspend actions and resume them after completing another unexpected action. Consequently, performance in task interruption situations relies on attention which is responsible for the control of actions during task-interruption situations.

Norman and Shallice (1986) proposed a model on the attentional control of actions. A basic notion in their model is that actions are represented in the cognitive system as action schemes. Action schemes are controlled by two mechanisms—namely, a contention scheduling mechanism and a supervisory attentional system (i.e., SAS). Contention scheduling governs behavior in routine situations. It selects relevant action schemes according to environmental cues and learnt habits. Moreover, it prevents incompatible schemes from controlling behavior by lateral inhibition. Thus, contention scheduling is an automatic conflict resolution process. In contrast, the SAS governs behavior in nonroutine situations and when action plans have to be modified. The SAS is an executive control system which monitors and controls the contention scheduling mechanism to allow for goal-directed behavior. Specifically, the SAS creates new action schemes and controls the activation and selection of action schemes by increasing and decreasing their activation. The SAS may contribute to performance in task-interruption situations because in such situations action plans have to be modified and/or new action schemes have to be developed.

Moreover, the negative effects of task interruptions on human performance have been discussed in research on prospective memory. Since in task-interruption situations, we have to form the intention to resume the primary task, task-interruption situations create a prospective memory task (e.g., Dodhia & Dismukes, 2009; Einstein et al., 2005). The intention to resume the primary task has to be maintained active in working memory, and the environment has to be monitored for cues that signal an opprtunity to exceute the intention. Such attentional and working memory processes are assumed to rely on limited cognitive resources (e.g., Smith, 2003). The recall of the intention to resume to primary task imposes demands on prospective memory which may be reflected by the resumption time.

The SAS model by Norman and Shallice (1986) and the prospectice memory view can explain numerous findings in task-interruption research by relating task-interruption effects to attentional control and working memory processes. Note, however, that these models were not originally developed to account for the effects of task-interruptions on human performance.

A further influential model on the cognitive basis of resumption costs is the memory for goals model (i.e., MFG model; Altmann & Trafton, 2002, 2015). According to this model, the processing of a task requires the retrieval of its goal (i.e., “mental representation of an intention to accomplish a task”; Altmann & Trafton, 2002, p. 39) from memory. The model states that goals have associated activation levels and that behavior is controlled by the goal with the highest current activation. Once retrieved, the activation of a goal is assumed to decrease gradually over time. The residual activation of old goals, thus, leads to interference when a new goal is retrieved from memory. If the activation level of the target goal is below the activation level of the most active nontarget goal, the distractor goal with the highest activation level instead of the target goal is sampled and guides actions.

Hence, the MFG model accounts for resumption costs by assuming that the activation of the primary task goal is subject to decay during the processing of the secondary task. To retrieve the primary task goal, a time-consuming reactivation of the primary task goal is required. Consequently, in the MFG model, resumption costs reflect cognitive processes related to the reactivation of a task goal that is necessary due to goal representation decay.

An alternative explanation of resumption costs might be that the primary task goal is inhibited when switching to the secondary task. When switching back to the primary task, the inhibitory aftermath has to be overcome, leading to resumption costs. Residual task inhibition as a source of interference during the resumption process has, however, not been in the focus of research attention.

In contrast to task-interruption research, the notion of task inhibition has been extensively examined in studies on task switching (see, e.g., Koch et al., 2010, for a review; see also Declerck & Koch, 2022, for the role of inhibition in bilingual language control). In such studies, subjects typically perform two (or more) simple categorization tasks (e.g., categorizing digits as odd or even) on a trial-by-trial basis (for reviews, see, e.g., Kiesel et al., 2010; Monsell, 2003; Vandierendonck et al., 2010). The sequence of the tasks is manipulated, resulting in *n* − 1 switch trials and *n* − 1 repetition trials. In *n* − 1 switch trials, the task in a given trial differs from the task performed in the previous trial, whereas in *n* − 1 repetition trials, the task is identical to that performed in the previous trial. A well-documented finding is that performance is worse in *n* − 1 switch trials than in *n* − 1 repetition trials, reflecting *n* − 1 task switch costs (e.g., Hirsch et al., 2018; for recent reviews, see, e.g., Koch & Kiesel, 2022; Koch et al., 2018).

Several models have been put forward to account for *n* − 1 switch costs. Generally, these models suggest that an abstract mental representation of a task, so-called task-set, has to be activated in working memory, to perform a task (e.g., Meiran, 1996; Rogers & Monsell, 1995). A task set is assumed to comprise various components necessary to proceed from stimulus encoding to responding, including information on task-relevant stimuli and responses, as well as on the corresponding stimulus–response mappings (for a detailed discussion on this concept, see, e.g., Kiesel et al., 2010; Vandierendonck et al., 2010).

Some models state that *n* − 1 switch costs reflect time-consuming and error-prone cognitive control processes responsible for reconfiguring the cognitive system appropriately for a new task (i.e., task-set reconfiguration models; e.g., Meiran, 1996; Rogers & Monsell, 1995). Other models assume that *n* − 1 switch costs are the result of automatic carryover effects of the preceding task (i.e., proactive interference models; e.g., Allport et al., 1994; Allport & Wylie, 1999, 2000). In particular, it is assumed that once a task-set has been activated, the activation persists even after task execution. In *n* − 1 switch trials, this persisting activation (positively) primes the currently irrelevant task set, leading to interference between the currently relevant and the previous task sets. Interference is additionally elicited by the prior inhibition of the currently relevant task set, which leads to negative priming of this task set when it needs to be re-activated.

Evidence that task inhibition can contribute to interference between tasks comes from task-switching studies using the backward inhibition paradigm (Mayr & Keele, 2000; for a review on inhibition, see, e.g., Koch et al., 2010). In this paradigm, subjects switch between three tasks (e.g., tasks A, B, & C) and performance is analyzed in *n* − 2 switch trials and *n* − 2 repetition trials. In *n* − 2 switch trials, the task in a given trial has not been performed in the preceding two trials (e.g., AB*C*), whereas in *n* − 2 repetition trials, a task re-occurs after an intervening trial (e.g., CB*C*). A typical finding with this paradigm is that performance is worse in *n* − 2 repetition trials than in *n* − 2 switch trials, reflecting *n* − 2 repetition costs.

These costs are interpreted as a behavioral marker of task inhibition. More precisely, it is hypothesized that a task switch requires the inhibition of the task set of the previously performed task, and that this inhibition has to be overcome, when switching back to this task.

In task-interruption research, residual inhibition of task sets (or task goals) has, to the best of our knowledge, not yet been taken into consideration as an explanatory concept for the interference during the resumption of the primary task. For instance, in the MFG model, which can account for numerous task-interruption effects, task interference is conceptualized only in terms of activation and its decay rather than in terms of inhibition. Other influential models, such as the time-based resource-sharing model (e.g., Barrouillet et al., 2004; Plancher & Barouillet, 2013) and the memory for problem states model (e.g., Barrouillet et al., 2004; Plancher & Barouillet, 2013; Borst et al., 2015), are also decay-based activation models which do not refer to inihibition.

## The present study

Since task inhibition has not yet received much attention in task-interruption research, it is an open question as to whether inhibition contributes to the interference between the secondary task and the primary task during the resuming process. In the present study, we addressed this novel question in three experiments. In all experiments, we implemented the backward inhibition paradigm into a task-interruption setting with a procedural task as primary tasks. By employing a procedural task, we had control over the sequence of the subtasks performed in the primary task. This allowed us to manipulate the *n* − 2 subtask sequence in the primary task and to analyze the role of residual inhibition of the last subtask performed before the interruption (i.e., last preinterruption subtask). In Experiment 1 and Experiment 2, the primary tasks consisted of three subtasks. The experiments differed in the extent of response-set overlap between the primary task and the secondary task. In Experiment 3, we examined the role of residual inhibition with a primary task comprising a sequence of four subtasks instead of three subtasks.

## Experiment 1

To explore whether residual inhibition of the last preinterruption subtask of the primary task contributes to the interference between the secondary and the primary task during the resumption process, we used two different procedural tasks consisting of a predefined order of multiple subtasks (e.g., Subtasks A, B, C) as primary tasks. As shown in Table 1, the sequence between the penultimate subtask and last subtask differed across the two primary tasks. There was either a subtask repetition across these subtasks (e.g., subtask *C* as penultimate subtask ➔ subtask *C* as last subtask) or a subtask switch (e.g., subtask *B* as penultimate subtask ➔ subtask *C* as last subtask). Thus, the primary tasks differed with regard to whether their last subtask was a *n* − 1 repetition subtask or a *n* − 1 switch subtask (i.e., relative to the penultimate subtask; e.g., A*CC* vs. A*BC*).

**Table 1:** Subtask sequences for the last subtask in noninterrupted and interrupted primary tasks

	*n* − 1 sequence in noninterrupted primary tasks	*n* − 2 sequence in interrupted primary tasks
Repetition sequences	A*BB*	A*B* interruption task *B*
Switch sequences	A*CB*	A*C* interruption task *B*

In some primary tasks, a task-interruption occurred before the last subtask of the primary task, changing the last subtask of noninterrupted primary tasks from a *n* − 1 repetition subtask to a *n* − 2 repetition subtask (e.g., subtask *C* as penultimate subtask → secondary task → subtask *C* as last subtask) and the *n* − 1 switch subtask to a *n* − 2 switch subtask (e.g., subtask *B* as penultimate subtask → secondary task → subtask *C* as last subtask).

We predicted that performance in the last subtask of the primary task is worse when a task interruption is performed before this subtask than when the last subtask is executed in a noninterrupted primary task. Moreover, in line with findings on task switching (for a review, see, e.g., Koch et al., 2018), we hypothesized that in noninterrupted primary tasks, the performance in the last subtask is worse for primary tasks with a subtask switch sequence than for primary tasks with a subtask repetition sequence, reflecting *n* − 1 subtask switch costs. For interrupted primary tasks, our prediction was that the performance in the last subtask is worse for primary tasks with a *n* − 2 repetition subtask as last subtask than for primary tasks with a *n* − 2 switch subtask as last subtask, resulting in *n* − 2 subtask repetition costs. Our rationale was as follows: If the subtask just performed before the interruption is inhibited when switching to the secondary task, the performance in the first subtask after the interruption (i.e., last subtask of the primary task) should be worse if this subtask is a *n* − 2 repetition subtask (i.e., a switch to the previously inhibited subtask is required) than if it is a *n* − 2 switch subtask. This *n* − 2 repetition cost would indicate that a part of the resumption cost often observed in task-interruption research is attributable to the need for overcoming the inhibition of the last preinterruption subtask.

Note that it would be sufficient to use a primary task consisting of two subtasks, in order to examine whether the last subtasks performed before the interruption is inhibited when switching to the interruption task. However, since we were interested in both sequence errors and nonsequence errors in Experiment 1, we used three subtasks to increase the memory demands for the primary task.

### Method

#### Participants

Fifty subjects participated in this preregistered experiment (41 women, age range: 18–28 years; *M* = 21.6 years; https://osf.io/f9j24). All participants gave written informed consent before the experiment. They received partial course credit and had normal or corrected-to-normal vision. All experiments of the present study were approved by the internal ethics committee at RWTH Aachen University (2020_019_FB07_RWTH Aachen) and their raw data is available at 10.23668/psycharchives.12938.

Note that a priori sample size estimation using G*Power (Faul et al., 2007) revealed that 44 participants are needed to detect a medium-sized effect (*d*_z_ = 0.5; here: subtask sequence in interrupted primary tasks) with a statistical power of 0.9. Due to reasons of counterbalancing the subtask order and the response keys, we planned to test 48 participants. Since we are not aware of other studies on *n* − 2 subtask sequence effects in task-interruption settings, we did not estimate the effect size for *n* − 2 subtask repetition costs after interruptions based on previous studies. We chose, however, an effect size which is in the range of the effect sizes reported for task-interruption effects (e.g., Altmann et al., 2017; Monk et al., 2008). In line with the description of the outlier and accuracy criteria presented in the preregistration, we excluded the data of two participants. To acquire complete counterbalancing, we tested two further participants and replaced the excluded data.

#### Stimuli, tasks, and responses

The experiment was programmed with the Gorilla Experiment Builder (www.gorilla.sc) and conducted online. The stimulus material comprised a white fixation cross (font size: 12.5 viewport height, vh; meaning 12.5% of the viewport height) and yellow and blue digits from 1 to 9, except 5, which were presented in Arial font in the centre of a black screen. The digits were used for both the primary and the secondary task.

The primary task consisted of three subtasks which had to be performed in a predefined order. In each subtask, we presented a single digit in the font size of 12.5vh. The subtasks were to decide whether the digit was presented in yellow or blue color (Subtask A), whether it was even or odd (Subtask B), or whether it was smaller or larger than 5 (Subtask C).

Each subtask was mapped to a different pair of response keys and fingers. Subjects performed the first subtask of the primary task with their little fingers, by pressing the “y” and the “,” keys of a QWERTZ keyboard. In the second subtask, they responded by pressing the “x” and “m” keys with their ring fingers, and in the third subtask, they used their middle fingers to press the “c” and “n” keys. The stimulus–response (S–R) mappings for the magnitude categorization subtask and parity categorization subtask were counterbalanced across subjects. Due to possible effects related to the mental number line (Dehaene et al., 1993), we counterbalanced the S–R mapping across subjects for all (sub)tasks requiring “numerical” decisions. For the color task, the S–R mapping was held constant because we did not assume an impact of the S–R mapping in this subtask on performance.

Each subject performed two different primary tasks. The order of the primary tasks was manipulated block-wise and was counterbalanced across subjects. As shown in Table 1, in both primary tasks, the relevant subtask switched from the first to the second subtask. Hence, the predefined sequences of both primary tasks were identical with regard to the sequence between the first subtask and the second subtask. The sequence between the second and the third subtasks, however, differed across the two primary tasks. For one primary task, there was a subtask repetition across the second subtask and the third subtask (e.g., A*CC*), whereas in the other primary task, the relevant subtask switched from the second subtask to the third subtask (e.g., ABC). Hence, the primary tasks differed with regard to whether the third subtask was a *n* − 1 repetition subtask or a *n* − 1 switch subtask. To control for effects related to the subtask order within a primary task, we used three different subtask orders and ensured that each subtask was presented once at each position in the subtask sequence (see Table 2). These subtask orders were counterbalanced across subjects.

**Table 2:** Subtask sequences of the primary task used in the present study

Sequences with a *n* − 1 repetition subtask as last subtask	Sequences with a *n* − 1 switch subtask as last subtask
Experiment 1 and Experiment 2	
ABB	ACB
BCC	BAC
CAA	CBA
Experiment 3	
ABBC	ACBA
BCCA	BACB
CAAB	CBAC

In the secondary task (i.e., interruption task), we employed a single digit in a font size of 37.5vh as stimulus. Thus, the font size was much larger than that of the stimuli for the primary task. The font size indicated that subjects had to perform the secondary task instead of the primary task. The secondary task was to decide if the digit is central (i.e., 3, 4, 6, 7) or peripheral (i.e., 1, 2, 8, 9) to 5. Subjects responded by pressing the “v” and “b” keys with their index fingers. The S–R mapping for the secondary task was counterbalanced across subjects. In interrupted primary tasks, the secondary task was introduced after the second subtask, changing the third subtask of a noninterrupted primary task from a *n* − 1 repetition subtask to a *n* − 2 repetition subtask and the *n* − 1 switch subtask to a *n* − 2 switch subtask (see Table 1).

#### Procedure

At the beginning of the experiment, the instructions for the first primary task appeared on the screen. Specifically, subjects were instructed to perform a primary task consisting of three subtasks which have to be performed in a predefined order. After presenting the stimulus–response mappings for each subtask of the primary task, the subjects were informed that the primary task will be occasionally interrupted by a secondary task, and they were presented with the S–R mappings for the secondary task. Moreover, they were instructed that after the completion of the secondary task, they should resume the primary task at the correct position. The instructions emphasized speed and accuracy for both the primary task and the secondary task.

Then, the subjects performed two practice blocks, followed by two experimental blocks. The first practice block included eight noninterrupted trials (i.e., primary tasks), whereas the second one comprised a random mixture of eight noninterrupted and 16 interrupted trials. In each experimental block, subjects performed a random mixture of 24 noninterrupted trials and 24 interrupted trials. Then, they received the instructions for the second primary task and performed the same number of blocks and trials like for the first primary task.

In both primary tasks*,* trials started with the presentation of a fixation cross for 1,500 ms, followed by the onset of a digit for the first subtask. Immediately after response execution, the first digit was replaced by a digit for the second subtask (i.e., response-stimulus-interval, RSI: 0 ms). In noninterrupted trials, the digit for the third subtask was presented immediately after the response execution for the second subtask, and in interrupted trials, instead of the digit for the third subtask, a digit in a larger font size was displayed for the secondary task. After response execution for the secondary task, the digit for the third subtask appeared on the screen. In practice blocks, information about the S–R mapping was presented in the upper left area of the screen for the duration of a primary task. In the case of an error, an error feedback (i.e., red cross) was provided for 500 ms, followed by the next digit. In experimental blocks, no reminder of the S–R mapping and no feedback were given.

Note that the secondary task was unpredictable and was not present in all primary tasks. The secondary task could occur only after the second subtask of the primary task, so that there was a perfect predictability for Subtask 1 and Subtask 2, but Subtask 3 could follow Subtask 2 immediately or only after the intervening secondary task.

#### Design

Performance in the third subtask of the primary task was analyzed based on a 2 × 2 repeated-measures design, with the within-subject independent variables interruption (interrupted vs. noninterrupted) and subtask sequence (subtask repetitions vs. switches). Note that we focused on the *n* − 1 subtask sequence in noninterrupted trials and on the *n* − 2 subtask sequence in interrupted trials (see Table 1). The dependent variables were reaction times (i.e., RT), sequence errors, and nonsequence errors.

### Results

Separate analyses of variance (ANOVAs) were run on mean RTs and error rates. We excluded practice trials, the first trial in each experimental block, and trials with an erroneous responses in Subtask 1, Subtask 2, and/or the secondary task from all analyses. As outliers, we defined trials with RTs in Subtask 3 deviating more than 3 standard deviations from a given participant’s mean per condition. In the RT analysis, only trials with correct responses in Subtask 3 were included. Moreover, we excluded the data of two participants due to an error rate of >78%, indicating that the participants did not understand the subtasks correctly.

#### RT

**Fig. 1:**
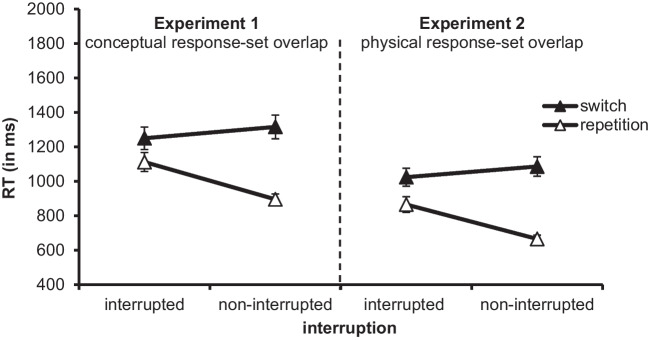
RT (in ms) in the third subtask of Experiment 1 and in Experiment 2 as a function of interruption (interrupted vs. noninterrupted primary tasks) and subtask sequence (in interrupted primary tasks: *n* − 2 switch vs. *n* − 2 repetition; in noninterrupted primary tasks: *n* − 1 switch vs. *n* − 1 repetition). Error bars represent the standard error of the mean

The ANOVA showed a main effect of interruption, *F*(1, 47) = 4.923, *p* = .031, η_p_^2^ = .095, reflecting higher RTs in the last subtask of interrupted primary tasks than in the last subtask of noninterrupted primary tasks (1181 ms vs. 1105 ms) and, thus, resumption costs of 76 ms. The main effect of subtask sequence, *F*(1, 47) = 24.974, *p* < .001, η_p_^2^ = .347, was significant, too. Participants responded more slowly when the last subtask of the primary task was a switch subtask than when it was a repetition subtask (1283 ms vs. 1003 ms). Moreover, the interaction of interruption and subtask sequence was significant, *F*(1, 47) = 44.632, *p* < .001, η_p_^2^ = .487, indicating that *n* − 1 subtask switch costs in noninterrupted trials were larger than *n* − 2 subtask switch costs in interrupted trials (421 ms vs. 138 ms; see Fig. 1). Post hoc one-tailed *t* tests showed that both the *n* − 1 subtask switch cost in noninterrupted primary tasks and the *n* − 2 subtask switch cost in interrupted primary tasks were significant, *t*(47) = 7.62, *p* < .001, *d*_z_ = 1.1, and *t*(47) = 2.161, *p* = .018, *d*_z_ = 0.312.

#### Error rates for nonsequence errors

For nonsequence errors, neither the main effect of interruption nor the main effect of subtask sequence were significant, both *F*s < 1 and *p*s > .486. The interaction of interruption and subtask sequence was not significant, too, *F*(1, 47) = 4.04, *p* = .05, η_p_^2^ = .079. However, there was a numerical trend towards *n* − 1 subtask switch costs in the last subtask of noninterrupted trials and *n* − 2 subtask repetition costs in the last subtask of interrupted trials (1.62% vs. −0.77%; see Table 3). Post hoc one-tailed *t* tests showed that in noninterrupted primary tasks, the *n* − 1 subtask switch cost was significant, *t*(47) = 2.023, *p* = .025, *d*_z_ = 0.292, whereas in interrupted primary tasks, the *n* − 2 subtask repetition cost was not significant, *t*(47) = 0.869, *p* = .195, *d*_z_ = 0.125.

**Table 3:** Mean error rates (in percentage; standard errors in parenthesis) in the third subtask of Experiment 1 and Experiment 2 as a function of interruption (interrupted vs. noninterrupted) and subtask sequence (repetition vs. switch)

	Noninterrupted primary tasks (*n* − 1 subtask sequence)	Interrupted primary tasks (*n* − 2 subtask sequence)
Experiment 1 (sequence of three subtasks with conceptual response-set overlap)		
Nonsequence errors		
Switch	4.8 (0.6)	3.6 (0.5)
Repetition	3.2 (0.5)	4.3 (0.8)
Sequence errors		
Switch	3.6 (0.6)	3.3 (0.6)
Repetition	2.9 (0.4)	3.8 (0.7)
Experiment 2 (sequence of three subtasks with physical response-set overlap)		
General errors		
Switch	6.6 (0.9)	6.4 (1.1)
Repetition	4.0 (0.6)	6.5 (1.1)

#### Error rates for sequence errors

For sequence errors, the main effects of interruption and subtask sequence, both *F*s < 1 and *p*s > .533, and the interaction of interruption and subtask sequence were not significant, *F*(1, 47) = 2.477, *p* = .122, η_p_^2^ = .05. Like for nonsequence errors, there was, however, a numerical trend towards *n* − 1 subtask switch costs in the last subtask of noninterrupted trials and a *n* − 2 subtask repetition cost in the last subtask of interrupted trials (0.73% vs. -0.54%; see Table 3). Post hoc one-tailed *t* tests showed that neither the *n* − 1 subtask switch cost nor the *n* − 2 subtask repetition cost were significant, *t*(47) = 1.089, *p* = .141, *d*_z_ = 0.157, and *t*(47) = 0.813, *p* = .211, *d*_z_ = 0.117.

### Discussion

In line with studies from the general task-switching domain (for a review, see, e.g., Koch et al., 2018), we found *n* − 1 subtask switch costs in the last subtask of noninterrupted primary tasks, indicating that switching between the subtasks of a primary task requires the reconfiguration of the cognitive system in accordance with a new subtask and/or the resolution of proactive interference elicited by the processing of a previous subtask. Most importantly, however, in contrast to our hypothesis about inhibitory subtask control, the RT data of interrupted primary tasks showed *n* − 2 switch costs instead of *n* − 2 repetition costs. Similarly, we did not find support for inhibition in the error data.

From task-switching research, there is evidence that inhibition occurs if there is strong interference between tasks (for a review, see, e.g., Koch et al., 2010). In Experiment 1, we used overlapping stimulus sets (i.e., same stimuli for all subtasks of the primary task and the secondary task). At the response level, different response keys were relevant for the secondary task and the subtasks of the primary task, but the responses overlapped at the left- and right-hand dimension. Consequently, inhibition might be required in task-interruption contexts with even stronger interference between the primary task and the secondary task.

## Experiment 2

In Experiment 2, we replicated Experiment 1 with physically overlapping responses. To this end, we used the same response keys for all subtasks of the primary task and the secondary task. Again, in addition to resumption costs, we predicted *n* − 1 subtask switch costs in noninterrupted primary tasks and *n* − 2 subtask repetition costs in interrupted primary tasks.

### Method

#### Participants

A new group of 50 subjects (44 women, age range: 18–29 years; *M* = 22.4 years) participated in the preregistered Experiment 2 (https://osf.io/t982q). They had normal, or correct-to-normal, vision and gave written informed consent prior to the study.

#### Stimuli, tasks, responses, procedure, and design

The stimuli, the tasks, the procedure, and the design were identical to those used in Experiment 1. However, as opposed to Experiment 1, participants responded in the secondary task and in all subtasks of the primary task by pressing the “v” and “b” keys with their index fingers. With this response set, it was not possible to distinguish between nonsequence errors and sequence errors, so that we analyzed general error rates as dependent variable in the ANOVA on the accuracy.

### Results

Using the same outlier criteria as in Experiment 1, we ran separate ANOVAs on mean RTs and error rates (see Fig. 1 and Table 3). For these ANOVAs, we excluded the data of two participants due to an excessive error rate of >69%.^1^

#### RT

For the RT data, there was a main effect of interruption, *F*(1, 47) = 7.835, *p* = .007, η_p_^2^ = .143, indicating that responses in the last subtask of the primary task were slower in interrupted primary tasks than in noninterrupted primary tasks (944 ms vs. 875 ms), reflecting resumption costs of 69 ms. Moreover, the main effect of subtask sequence was significant, *F*(1, 47) = 50.997, *p* < .001, η_p_^2^ = .52. Participants responded more slowly when the last subtask of the primary task was a switch subtask than when it was a repetition subtask (1055 ms vs. 765 ms), resulting in subtask switch costs. The interaction of interruption and subtask sequence was significant, too, *F*(1, 47) = 39.671, *p* < .001, η_p_^2^ = .458. The *n* − 1 subtask switch cost in noninterrupted primary tasks was greater than the *n* − 2 subtask switch cost in interrupted primary tasks (421 ms vs. 159 ms). In line with Experiment 1, post hoc one-tailed *t* tests showed that both the *n* − 1 subtask switch cost and the *n* − 2 subtask switch cost were significant, *t*(47) = 9.227, *p* < .001, *d*_z_ = 1.339, and *t*(47) = 3.477, *p* < .001, *d*_z_ = 0.502.

#### Error rates

For the accuracy data, the interaction of interruption and subtask sequence was significant, *F*(1, 47) = 5.289, *p* = .026, η_p_^2^ = .101, reflecting *n* − 1 subtask switch costs of 2.6% in noninterrupted primary tasks and *n* − 2 subtask repetition costs of 0.13% in interrupted primary tasks. As indicated by post hoc one-tailed *t* tests, the *n* − 1 subtask switch costs was significant, *t*(47) = 3.298, *p* = .001, *d*_z_ = 0.476, whereas the *n* − 2 subtask repetition cost was not significant, *t*(47) = 0.112, *p* = .456, *d*_z_ = 0.016. The main effects of interruption and subtask sequence were not significant, *F*(1, 47) = 2.902, *p* = .095, η_p_^2^ = .058, and *F*(1, 47) = 2.542, *p* = .118, η_p_^2^ = .051. Descriptively, there was, however, a trend towards more errors in the last subtask of interrupted primary tasks than in the last subtask of noninterrupted primary tasks (6.45% vs. 5.29%) and in switch sequences than in repetition sequences (6.49% vs. 5.25%).

### Discussion

Replicating the findings of Experiment 1, we observed that task-interruptions result in a performance decline in the subtask after an interruption and that switching between subtasks in noninterrupted primary tasks leads to *n* − 1 subtask switch costs. Moreover, we again observed *n* − 2 subtask switch costs. Thus, even with increased between-task interference due to physically overlapping response-sets, we found no evidence for the inhibition of the last preinterruption subtask of the primary task.

## Experiment 3

In Experiment 1 and Experiment 2, in contrast to our prediction, we found no evidence that residual inhibition of the last preinterruption subtask contributes to resumption costs. However, in these experiments, the subtask switch and the subtask repetition sequences of the primary task differed in the number of subtasks which participants had to maintain in working memory. This is because in primary tasks with a subtask switch sequence, subjects had to perform three different subtasks (e.g., ABC), whereas in those with a subtask repetition sequence, they had to perform two different subtasks (e.g., BBC; see Table 1). Thus, based on these experiments, we could not exclude that the observed *n* − 2 subtask switch cost was due to differences in the working memory load across the subtask sequences.^2^

To isolate the effects of the *n* − 2 subtask sequence manipulation in Experiment 3, we combined the three subtasks used in the previous experiments to subtask sequences consisting of four subtasks and replicated Experiment 2 with primary tasks comprising these new subtask sequences. In both the primary task with subtask switch sequences and the primary task with subtask repetition sequences, subjects performed three different subtasks. In primary tasks with a subtask switch sequence, the new, fourth subtask was identical to the first subtask (e.g., ABCA which was previously ABC). In primary tasks with a subtask repetition sequence, we added the previously unused subtask as fourth subtask (e.g., ACCB which was previously ACC). Note that the first three subtasks in both subtasks sequences were identical to those employed in Experiment 1 and Experiment 2. By adding the fourth subtask, we were able to ensure that in both subtask repetition sequences and subtask switch sequences three different subtasks had to be maintained in working memory. Using this modified primary tasks, we examined the effects of the *n* − 2 subtask sequence manipulation in an experimental setting with a constant working memory load across primary tasks.

### Method

#### Participants

We tested a new group of 48 subjects (36 women, age range: 18–34 years; *M* = 23.1 years) with normal, or correct-to-normal, vision. All participants gave written informed consent prior to the experiment.

#### Stimuli, tasks, responses, procedure, and design

We used the same stimuli, tasks, procedure, and design like in Experiment 2, except that the primary tasks consisted of a predefined sequence of four subtasks.

### Results

**Fig. 2:**
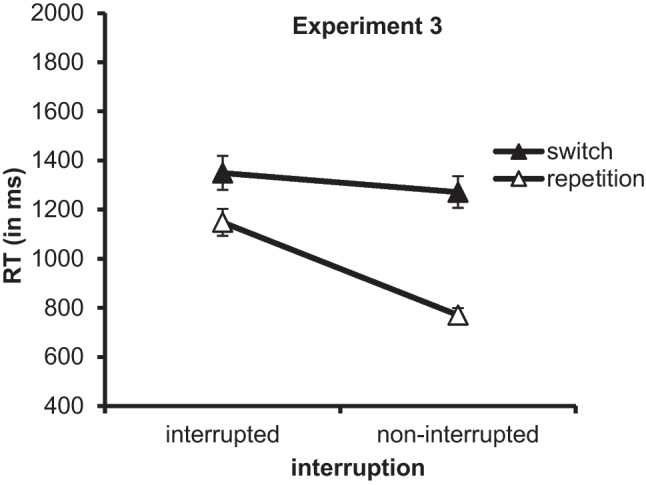
RT (in ms) in the third subtask of Experiment 3 as a function of interruption (interrupted vs. noninterrupted primary tasks) and subtask sequence (in interrupted primary tasks: *n* − 2 switch vs. *n* − 2 repetition; in noninterrupted primary tasks: *n* − 1 switch vs. *n* − 1 repetition). Error bars represent the standard error of the mean

We used the same outlier criteria like in the previous experiments and ran separate ANOVAs on mean RTs and error rates (see Fig. 2 and Table 4).

**Table 4:** Mean error rates (in percentage; standard errors in parenthesis) in the third subtask of Experiment 3 (sequence of four subtasks with physical response-set overlap) as a function of interruption (interrupted vs. noninterrupted) and subtask sequence (repetition vs. switch)

	Noninterrupted primary tasks (*n* − 1 subtask sequence)	Interrupted primary tasks (*n* − 2 subtask sequence)
Experiment 3 (sequence of four subtasks with physical response-set overlap)		
General errors		
Switch	2.1 (1.1)	2.9 (0.9)
Repetition	4.6 (1.0)	3.4 (0.9)

#### RT

The ANOVA on the RT data showed a significant main effect of interruption, indicating that RTs in the third subtask of the primary tasks were higher when there was an interruption before the third subtask than when there was no interruption (1,249 ms vs. 1,020 ms), *F*(1, 47) = 37.83, *p* < .001, η_p_^2^ = .446. Thus, there were resumption costs of 229 ms. The main effect of subtask sequence was significant, too, *F*(1, 47) = 47.21, *p* < .001, η_p_^2^ = .501. Participants responded more slowly when the third subtask was a switch subtask than when it was a repetition subtask (1310 ms vs. 959 ms). Finally, the ANOVA yielded a significant interaction of interruption and subtask sequence, *F*(1, 47) = 24.527, *p* < .001, η_p_^2^ = .343, reflecting *n* − 1 subtask switch costs of 501 ms in noninterrupted primary tasks and *n* − 2 subtask switch costs of 201 ms in interrupted primary tasks. Post hoc one-tailed *t* tests showed that both the *n* − 1 subtask switch cost and the *n* − 2 subtask switch cost were significant, *t*(47) = 9.817, *p* < .001, *d*_z_ = 1.417, and *t*(47) = 3.014, *p* = .002, *d*_z_ = 0.435.

#### Error rates

For the ANOVA on the error rates, there was a significant main effect of subtask sequence, *F*(1, 47) = 9.305, *p* = .004, η_p_^2^ = .165, reflecting more errors in the third subtask when the primary task comprises a subtask switch sequence than when it comprises a subtask repetition sequence (4% vs. 2.5%). The main effect of interruption was not significant, *F* < 1 and *p* < .552. However, the interaction of interruption and subtask sequence was significant, *F*(1, 47) = 5.194, *p* = .027, η_p_^2^ = .1, indicating *n* − 1 subtask switch costs of 2.5% in noninterrupted primary tasks and *n* − 2 subtask switch costs of 0.5% in interrupted primary tasks. Post hoc one-tailed *t* tests showed that the *n* − 1 subtask switch cost in noninterrupted trials was significant, *t*(47) = 3.299, *p* < .001, *d*_z_ = 0.476, whereas the *n* − 2 subtask switch cost in interrupted trials was not significant, *t*(47) = 0.921, *p* = .181, *d*_z_ = 0.131.

### Discussion

When controlling for differences in working memory load across primary tasks comprising subtasks switch sequences and primary tasks comprising subtask repetition sequences, we replicated the findings of Experiment 1 and Experiment 2. We observed resumption costs, *n* − 1 subtask switch costs, and *n* − 2 subtask switch costs. Thus, like in the previous experiments, we found no evidence for the inhibition of the last preinterruption subtask of the primary task.

## General discussion

The aim of the present study was to investigate the novel question of whether residual inhibition of the last preinterruption subtask leads to interference during the resumption of the primary task. To this end, we conducted three experiments in which we implemented the backward inhibition paradigm often used in task-switching studies into a task-interruption context. In Experiment 1 and Experiment 2, we employed a predefined sequence of three subtasks. The experiments differed with regard to the extent of response-set overlap between the primary task and the secondary task. Whereas the responses of the primary task and the secondary task overlapped conceptually in Experiment 1, the responses overlapped physically in Experiment 2, thereby increasing the interference between the primary task and the secondary task. In Experiment 3, we replicated Experiment 2 with a primary task comprising a predefined sequence of four subtasks to exclude differences in working memory load across subtask switch and subtask repetition sequences as an alternative explanation for the finding of *n* − 2 switch costs. In all experiments, we observed resumption costs. Moreover, we found *n* − 1 switch costs in noninterrupted primary tasks and *n* − 2 switch costs in interrupted primary tasks.

### Resumption costs

In line with previous studies which used procedural tasks as primary task (e.g., UNRAVEL task by Altmann et al., 2014; see also Radović & Manzey, 2022), we found that task interruptions prolong the processing time of the subtask performed immediately after the interruption, reflecting resumption costs (for reviews, see, e.g., Couffe & Michael, 2017; Hirsch et al., 2022). This indicates that the resumption of the primary task relies on time-consuming cognitive processes like proposed in the SAS model, the prospective memory view, and the memory for goals model. Concerning the vulnerability to errors, we found no effect of task interruptions on nonsequence errors and sequence errors. Consequently, it remains unclear whether the effect of task interruptions on performance is due to attentional processes, memory processes, or both.

Some previous task-interruptions studies found an increase in error rates after an interruption relative to noninterrupted primary tasks (e.g., Altmann et al., 2014, 2017). In these studies, subjects performed primary tasks consisting of a predefined sequence of more than four subtasks (e.g., seven subtasks in Altmann et al. 2014). Thus, a possible reason why we did not find a negative effect of task interruptions on accuracy in the present study might be that demands on the place-keeping ability might be somewhat lower in the present task-interruption paradigm with primary tasks consisting of three or four subtasks than the demands in other studies. The finding of substantial resumption costs in the RT data of all three experiments indicates that our task-interruption paradigm with a procedural primary task of three to four subtasks is well suited for exploring the cognitive basis of performance costs in task-interruption situations, but the paradigm might be more sensitive for task-interruption effects in processing speed than in accuracy.

### The role of residual inhibition of the primary task in resumption costs

In all experiments, we observed *n* − 1 subtask switch costs in the third subtask of noninterrupted primary tasks. These costs provide evidence that a reconfiguration of the cognitive system for a new subtask and/or the resolution of proactive interference is required, when switching between the subtasks of the primary task (for a desciption of similar models in task-switching research, see Koch & Kiesel, 2022). Since we did not manipulate the *n* − 2 subtask sequence in noninterrupted primary tasks, we, however, cannot answer the question whether switching between subtasks of the primary tasks requires inhibitory control.

In contrast to noninterrupted primary tasks, there was a *n* − 2 subtask sequence manipulation in interrupted primary tasks. In three experiments, these manipulation resulted in *n* − 2 subtask switch costs in the subtask following an interruption. Importantly, as indicated by Experiment 3, the worse performance in *n* − 2 subtask switch sequences relative to *n* − 2 subtask repetition sequences was not fully attributable to the higher working memory load in *n* − 2 subtask switch sequences than in *n* − 2 subtask repetition sequences.

For the general task-switching domain, Grange et al. (2013) demonstrated based on computational modelling that the absence of *n* − 2 sequence effects cannot rule out the existence of task inhibition because inhibition might decay during the processing of another task. The existence of *n* − 2 switch costs is, however, according to the findings of this study, a strong indicator that a task performed before an intervening task was not inhibited.

Consequently, we can draw the following two conclusions regarding the *n* − 2 subtask switch costs observed in the present study. First, the activation of the last preinterruption subtask persists during the secondary-task processing and this persisting activation results in residual positive priming of the last preinterruption subtask during the resumption process. Second, during the resumption of the primary task, positive priming of the last preinterruption subtask was at least stronger than the effects of inhibition.

Note that we cannot rule out that inhibition plays no role at all in task-interruption contexts. This is because positive priming and inhibition might operate at different levels. In task-interruption situations with procedural primary tasks, there are at least two types of cognitive representations relevant (Altmann & Trafton, 2015). First, to execute a specific subtask, the corresponding cognitive representation of this subtask has to be activated in working memory. This subtask representation organizes operations like stimulus interpretation and response selection. Second, a cognitive representation including information on the subtask sequence is required to perform the primary task. This “superordinate” representation enables participants to perform the subtasks of the primary task in the correct order. Thus, inhibition might operate at the level of individual subtask representations or at the level of “superordinate” primary task representations. Task-switching studies in which subjects switch between the S–R mappings of completed, discrete tasks suggest that subjects inhibit the recently performed task representation when switching to a new task. In contrast, research on superordinate representations indicates that this type of cognitive representations is not inhibited (e.g., Hirsch et al., 2017). In the present study, we focused on the last subtask performed before the interruption and, thus, on subtask level.

The conclusion that residual inhibition of the primary task does not substantially contribute to resumption costs is in line with the model on the attentional control of actions, the prospective memory view, and the MFG model. In the model on the attentional control of actions by Norman and Shallice (1986), the *n* − 2 switch cost after an interruption suggests that the action schema for the primary task is not inhibited by the SAS. Thus, the SAS might maintain the schema for the primary task on a high activation level during the processing of the secondary task. According to the prospective memory view, the intention to resume the primary task is maintained in working memory instead of being inhibited during the processing of the secondary task. The finding is also consistent with the MFG model. Like other influential models in task-interruption research (see, e.g., time-based resource model by Barrouillet et al., 2004; Plancher & Barouillet, 2013; memory for problem states model by Borst et al., 2015), the MFG model conceptualizes the interference between the primary task and secondary task during the resumption process in terms of activation decay without any involvement of primary-task inhibition.

Based on this model, the *n* − 2 subtask switch cost observed in the present study can be accounted for as follows: Repeating the subtask just performed before the interruption is beneficial because there is a residual activation of this subtask when resuming the primary task. Due to this residual activation less time is needed for the activation strengthening of this subtask after the interruption. In situations in which a new subtask is performed after the interruption, there should be no or weaker residual activation of this subtask after the interruption, so that more time is needed for the activation strengthening.

Task inhibition has been shown to play a crucial role in task-switching situations that require the switching back and forth between the stimulus–response mappings of completed discrete tasks. For the resumption process, however, inhibition seems to play no crucial role. This is at least the case when considering the *n* − 2 repetition cost as an empirical marker for residual inhibition. Thus, *n* − 2 repetition costs could not be replicated outside the specific task-switching context in which they are usually investigated. However, there are crucial differences between our task-interruption study and typical *n* − 2 backward inhibition studies in task-switching research. We do not know which of these differences might be critical to observe the relative difference of inhibitory after-effects measured with *n* − 2 repetition costs.

In our study, *n* − 2 subtask repetitions occurred only if there was a task interruption. Trials without task interruptions included immediate (i.e., *n* − 1) subtask repetitions. Philipp and Koch (2006) showed that *n* − 2 repetition costs are small if there are immediate task repetitions, indicating that our conditions might be not favorable to observe *n* − 2 repetition costs at the subtask level.

A further potential reason for this discrepancy across task-switching and task-interruption contexts might be that task-interruptions have strong memory demands. To ensure a correct resumption of the primary task after the secondary task, subjects have to maintain the last subtask performed before the interruption or the first subtask to-be-performed after the interruption during the processing of the secondary task. Thus, it might be costly to inhibit the last subtask performed before the interruption because this subtask can help to specify the position in the primary task after the completion of the secondary task (see also Ratwani & Trafton, 2010).

### Methodological considerations

Even though our task-interruption paradigm shares some similarities with task-switching paradigms, there are crucial differences between them at both the methodological and the theoretical level. At the methodological level, in task-switching experiments with a random task sequence, subjects switch between the stimulus–response mappings of completed discrete tasks (see Monk et al., 2008). In contrast, in our task-interruption paradigm and all task-interruption studies with a procedural task as primary task (e.g., UNRAVEL task by Altmann et al., 2014, or WINDA task by Kopacz et al., 2019), subjects are instructed to perform a more complex single task which is made up of several subtasks which have to be performed in a specific order. In task-switching experiments using the alternating runs paradigm, subjects also perform a fixed sequence of tasks. This is because they are instructed two switch the task after a certain number of task repetitions (e.g., after each second task repetition; e.g., AABB). However, in contrast to our task-interruption paradigm, in the alternating-runs paradigm, subjects are not instructed that there is a main task consisting of a predefined order of subtasks and, most importantly, no additional task is inserted unpredictably into the fixed sequence. Thus, there is no interruption task in the alternating-runs paradigm, and the task sequence is always predictable. Moreover, in the alternating runs paradigm, there are no clear starting and end points of a task sequence. Rather, the sequence is repeated over the entire block. In our task-interruption paradigm, there is a fixation cross before a new primary task, indicating that the previous primary task is completed and a new primary tasks is beginning.

At the theoretical level, in task-switching experiments, the emphasis is on the cognitive mechanisms underlying task-switching performance. In all task-interruption paradigms with a procedural primary task, subjects switch between (sub)tasks, too. However, in contrast to task-switching paradigms, there is additionally the need (1) to create a cognitive representation of the entire subtask sequence before starting to perform the primary task. Moreover, subjects (2) have to form an intention to resume the primary task and (3) have to maintain this intention, along with the processing state of the primary task, when shifting attention to the secondary task. Thus, the emphasis is not on the switching mechanism but on the cognitive processes allowing subjects to resume the primary task.

Moreover, one could argue that the resumption lag observed in the present study is due to the fact that in interrupted primary tasks subjects switch from a less often performed interruption task to Subtask 3, whereas in noninterrupted primary tasks, they switch from Subtask 2 that is performed in every primary task. This difference is the result of having a baseline condition without an interruption and is not avoidable in task-interruption situations.

A further aspect that needs to be considered when interpreting the results of this study is that the data was collected online. It has been shown that in online studies, the technology (e.g., software and hardware) used by the participants can influence the precision of the stimulus and response timing (e.g., Anwyl-Irvine et al., 2021). Such differences in timing might reduce data quality. In addition, factors such as noise and increased distraction can have an impact on data quality, (Segen et al., 2021). Therefore, it is important to replicate our study in an online setting with a greater number of trials and/or to confirm our findings in a laboratory study.

### Summary and conclusions

Taken together, the findings of the present study suggest that a switch to the secondary task is presumably not accompanied by the inhibition of the last preinterruption subtask. Consequently, residual inhibition of the primary task, at least at the level of subtasks, does not appear to contribute to the interference between the primary task and the secondary task during primary task resumption. This suggests that the subtask selection mechanism in procedural tasks is mainly supported by persisting activation that results in residual positive priming of the preinterruption. Note, however, that so far, the role of residual inhibition for resumption costs has hardly been in the focus of task-interruption research. Thus, more research is warranted to examine whether the findings observed in the present study are restricted to the specific experimental paradigm or whether they generalize to other task-interruption situations.

## Data Availability

The data sets generated during the current study are available in the PsychArchives (10.23668/psycharchives.12938). Moreover, Experiment 1 and Experiment 2 were preregistered prior data collection in open science framework (https://osf.io/f9j24 and https://osf.io/t982q).

## Footnotes

1 Note that we preregistered a further analysis. In this analysis, we planned to examine the *n* − 2 repetition cost as a function of the response-set overlap. However, since we did not find any significant *n* − 2 repetition costs, we did not conduct this between-experiment analysis.

2 Note that to examine the role of working memory load in Experiment 1 and Experiment 2, we analyzed whether the RTs in the first and second subtasks differed as a function of the subtask sequence. In Experiment 1, there was a numerical trend towards higher RTs for Subtask 1   in subtask switch sequences than in subtask repetition sequences (1,249 vs. 1,190 ms), *t*(47) = 1.149, one-tailed *p* = .128, *d*_z_ = 0.166. For Subtask 2, this effect was significant (1,188 ms in subtask switch sequences vs. 1,060 ms in subtask repetition sequences), *t*(47) = 3.77, *p* < .001, *d*_z_ = 0.544. In Experiment 2, RTs were higher in subtask switch sequences than in subtask repetition sequences for both Subtask 1 (1,098 ms vs. 1,022 ms), *t*(47) = 2.112, one-tailed *p* = .02, *d*_z_ = 0.305, and Subtask 2 (1,050 ms vs. 889 ms), *t*(47) = 3.953, one-tailed *p* < .001, *d* = 0.571. Thus, this analysis showed that working memory load was at least partly the reason for slower responses in subtask switch sequences than in subtask repetition sequences.
